# Biomechanical Evaluation of a Mandibular Spanning Plate Technique Compared to Standard Plating Techniques to Treat Mandibular Symphyseal Fractures

**DOI:** 10.1155/2015/569030

**Published:** 2015-11-16

**Authors:** Matthew Richardson, Jonathan Hayes, J. Randall Jordan, Aaron Puckett, Matthew Fort

**Affiliations:** ^1^University of Mississippi Medical Center, Department of Otolaryngology and Communicative Sciences, 2500 N. State Street, Jackson, MS 39216, USA; ^2^University of Mississippi Medical Center, Department of Biomedical Materials Science, 2500 N. State Street, Jackson, MS 39216, USA

## Abstract

*Purpose*. The purpose of this study is to compare the biomechanical behavior of the spanning reconstruction plate compared to standard plating techniques for mandibular symphyseal fractures.* Materials and Methods*. Twenty-five human mandible replicas were used. Five unaltered synthetic mandibles were used as controls. Four experimental groups of different reconstruction techniques with five in each group were tested. Each synthetic mandible was subjected to a splaying force applied to the mandibular angle by a mechanical testing unit until the construct failed. Peak load and stiffness were recorded. The peak load and stiffness were analyzed using ANOVA and the Tukey test at a confidence level of 95% (*P* < 0.05).* Results*. The two parallel plates' group showed statistically significant lower values for peak load and stiffness compared to all other groups. No statistically significant difference was found for peak load and stiffness between the control (C) group, lag screw (LS) group, and the spanning plate (SP1) group.* Conclusions*. The spanning reconstruction plate technique for fixation of mandibular symphyseal fractures showed similar mechanical behavior to the lag screw technique when subjected to splaying forces between the mandibular gonial angles and may be considered as an alternative technique when increased reconstructive strength is needed.

## 1. Introduction

**Figure 1:**
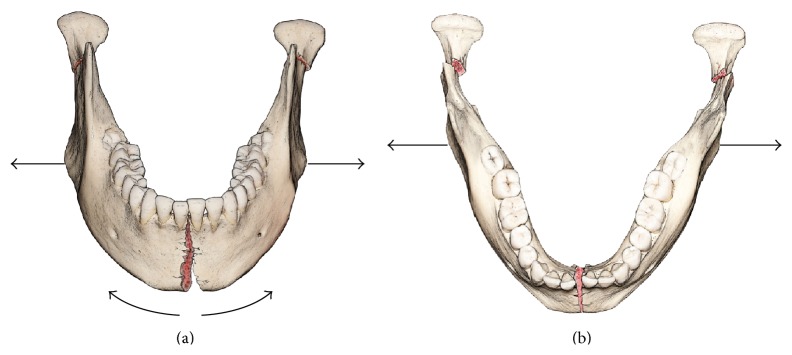
Illustration showing symphyseal fracture with bicondylar fractures and the resulting flaring (a) and widening (b) of the gonial angles.

The mandible is one of the most commonly fractured bones in the facial skeleton. Symphyseal and parasymphyseal fractures of the mandible have been reported to occur with a frequency of 9% to 57% [1, 2]. Treatment of mandible fractures is based on the restoration of form and function. This requires anatomic reduction of the mandible to its pretraumatic shape and proper fixation of the fracture to resist deformation. The anatomy of the mandible and vector of forces exerted by the suprahyoid, masseter, and temporalis muscles make symphysis fractures particularly problematic in this regard. When the muscles of mastication contract to bite and clench, the mandible is bent in a sagittal plane. There is bilateral torsion of the mandibular bodies resulting in bending at the symphyseal region. This in turn leads to compression at the superior margin of the symphysis and tension at the inferior margin. Late in the power stroke of biting and clenching, lateral transverse bending occurs and the bending moment increases from back to front to reach its maximum magnitude near the symphysis. This lateral bending produces compressive stress at the buccal cortex and tensile stress at the lingual surface [3, 4]. In a patient with a symphyseal fracture, this results in the hemimandible being splayed outward. This is especially true in the setting of a mandibular symphysis fracture associated with a unilateral or bilateral mandibular subcondylar fracture. The symphyseal mandibular fracture with bilateral condylar or subcondylar fractures is a somewhat common fracture pattern and often a very difficult reconstructive problem [5]. In this setting, there is no longer posterior stability provided by the temporomandibular articulation and the mandibular gonial angles are flared [6] (Figure 1).

The various fixation techniques of mandibular fractures have evolved over time based on the patient's needs and the most recent scientific and surgical advances. For over thirty years, the treatment of choice has been open reduction with stable internal fixation. The lag screw technique, which was first described in 1970, was more recently shown by Madsen et al. to be mechanically superior to other commonly used techniques including the two-plate techniques [7].

A spanning reconstruction plate between the inferior borders of the mandibular body positions the long axis of the plate parallel with the splaying forces on the mandible. This offers a theoretical mechanical advantage, but the biomechanical behavior of the spanning reconstruction plate used in conjunction with two parallel plates' system has never been evaluated. Therefore, the purpose of this study is to evaluate the biomechanical behavior of the spanning reconstruction plate and compare it to the lag screw technique and the two parallel plates' technique. The parameters evaluated were peak load and stiffness. Peak load is the load at which permanent deformation of the system begins. Stiffness is a parameter used to describe the force needed to achieve a certain deformation of a structure (stiffness = the slope of the force divided by deformation).

## 2. Materials and Methods

A total of 25 human mandible replicas (Sawbones Foam Cortical Shell Mandibles, Pacific Research Laboratories, Vashon, Washington) were used in this study. These synthetic replicas were chosen to eliminate the variation in geometry and mechanical properties of human bone. They have been shown to be appropriate maxillofacial human bone substitutes for testing the stability of rigid fixation techniques [8].

Fixation materials consisted of 4-hole 0.6 mm thick straight titanium miniplates with spacer (Stryker Maxillofacial, 55-06704), 4-hole straight 1.5 mm thick titanium mandible miniplates with spacer (Stryker Maxillofacial, 55-10505), 12-hole 0.6 mm thick straight titanium plates (Stryker Maxillofacial, 55-06724), 30 mm 2.0 mm diameter titanium lag screws (Stryker Maxillofacial, 53-20430), 1.7 mm titanium monocortical bone screws each being 4 mm long (Stryker Maxillofacial, 50-17004), 2.0 mm titanium monocortical bone screws each being 10 mm long (Stryker Maxillofacial, 50-17004), and 2.0 mm 3D rectangular plates, 3 × 2 holes (Stryker Maxillofacial, 55-10532).

### 2.1. Sample Preparation

Five synthetic mandibles were unaltered and served as controls. Twenty synthetic mandibles were divided into four experimental groups with five mandibles in each group. Each experimental synthetic mandible was split evenly at the midline between the mandibular central incisors. The cuts were made with alloy steel blade coupled to a hand piece and a customized jig with saw guide was used to ensure uniformity of cuts. Each experimental group was fixed with a different technique (Table 1). Each reconstruction was performed using a customized jig made from epoxy resin to ensure consistent positioning of both mandibular segments.

**Figure 2:**
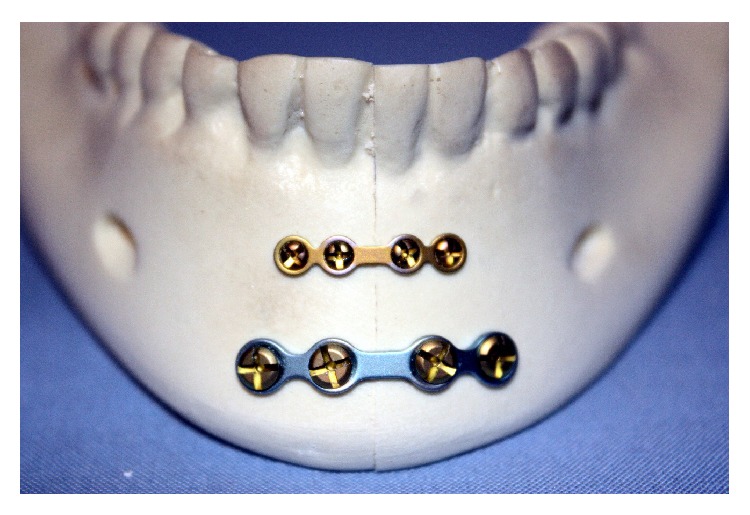
Two parallel plates (2PP).

**Figure 3:**
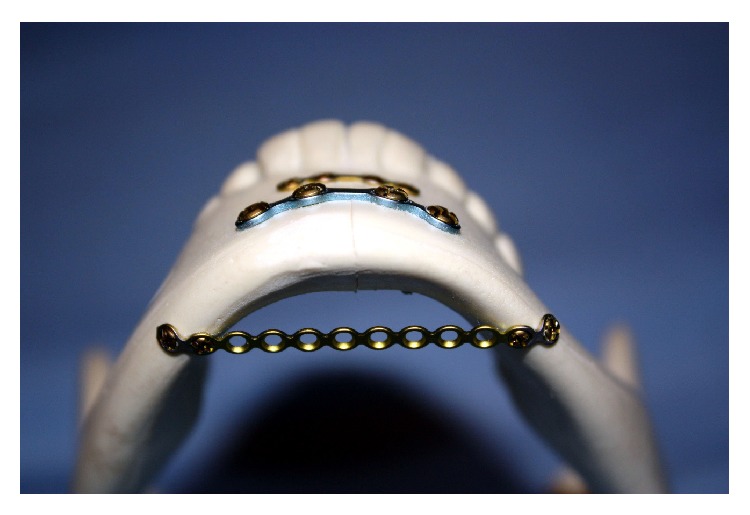
Two parallel plates plus spanning plate (SP1).

**Figure 4:**
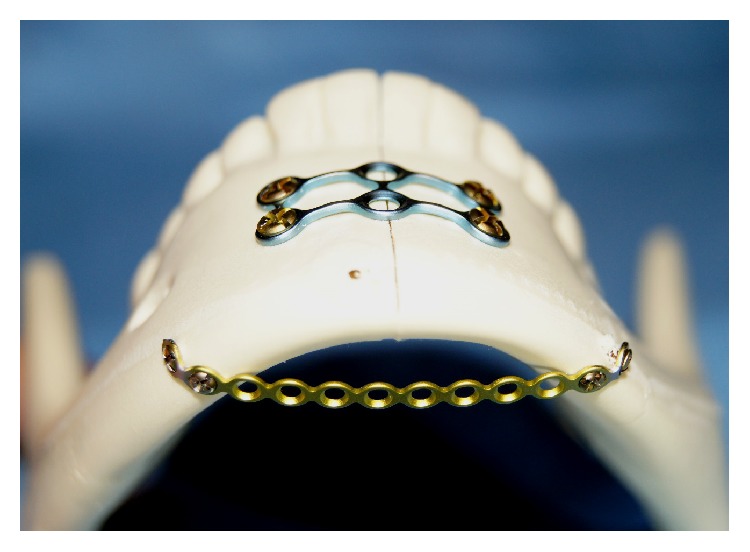
Ladder plate plus spanning plate (SP2).

**Figure 5:**
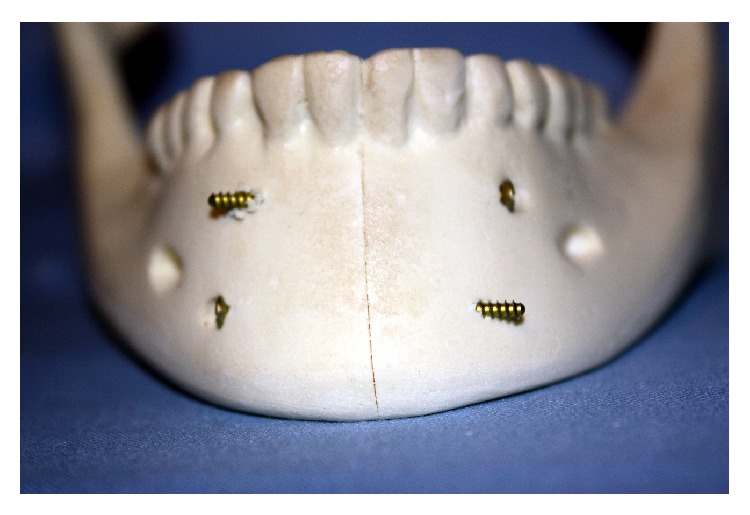
Lag screw (LS) group.

The two parallel plates' (2PP) group was fixed with a 4-hole 0.6 mm thick miniplate secured to the superior border with four 1.7 mm outer diameter screws each being 4 mm long. The inferior border was fixed with the larger 4-hole 1.5 mm thick mandibular miniplate and secured with four 2.0 mm outer diameter screws each being 10 mm long (Figure 2). The 2PP plus spanning plate (SP1) group was fixed in a similar fashion as the two parallel plates' group with the addition of a straight 12-hole 1.5 mm thick plate spanning between the inferior borders of the mandibular body. This plate was secured using four 1.7 mm outer diameter screws each being 4 mm long (Figure 3). The second-spanning plate (SP2) group was fixed in a similar fashion to SP1 group with respect to the spanning plate, but with a 6-hole (3 holes × 2 holes) ladder plate rather than two parallel plates (Figure 4). The lag screw (LS) group was fixed with two 30 mm long 2.0 mm diameter self-tapping screws by the lag screw technique (Figure 5).

### 2.2. Load Testing

**Figure 6:**
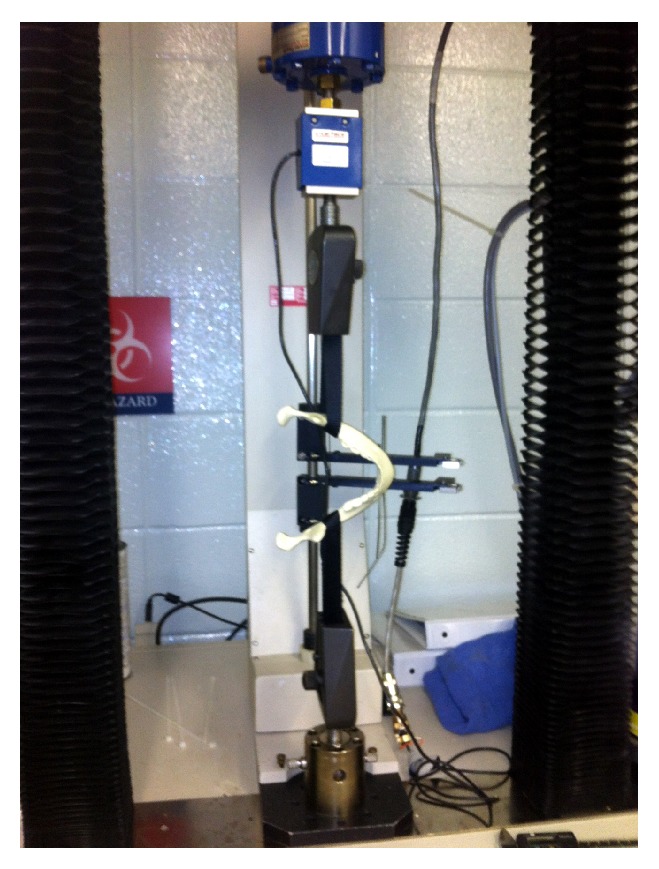
Sample positioned for mechanical testing in the Sintech 2/G servohydraulic materials testing unit.

Biomechanical testing was performed on each synthetic mandible properly prepared with the respective fixation method. Each mandible was tested only once. Five uncut mandibles served as controls to define the limitations of the substrate (synthetic mandible replica). Each sample was placed in a custom fabricated jig consisting of two heavy-duty nylon straps. Each nylon strap was folded onto itself to create a loop. One nylon strap loop was placed over the condylar and coronoid heads and seated at the angle of the mandible on each side. The nylon strap loops were attached to the vertical arms of the mechanical testing unit. Each construct was preloaded with 0.5 lbs to provide enough tension between nylon strap loops such that the mandible would be suspended between them. Vertical loads were created and measured with a Sintech 2/G (MTS Corporation, Minneapolis, MN) servohydraulic materials testing unit (Figure 6). The vector force was therefore lateral to each mandibular angle simulating the physiologic splaying forces on the mandible from the suprahyoid, masseter, and temporalis muscles. The servohydraulic testing unit developed a linear displacement at a rate of 10 mm per minute, and 250 lbs load cell measured the resultant force. Loading was continued up to mechanical failure of each construct. Data were captured and analyzed with the TestWorks 4 (MTS Corporation, Minneapolis, MN) software. Means were calculated for peak load and stiffness of each test group (Table 2). The peak load and stiffness were analyzed using ANOVA and the Tukey test at a confidence level of 95% (*P* < 0.05). Results between groups were compared for statistical significance using the Mann-Whitney test allowing for nonparametric data analysis (Table 3). A two-tailed *P* less than 0.01 was considered significant.

## 3. Results

After statistical analysis of values for peak load, the results showed a statistically lower value for the two parallel plates' (2PP) group compared to the spanning plate (SP1) group, the lag screw (LS) group, and the control (C) group. No significant difference was found between the spanning plate (SP1) group, the lag screw (LS) group, and the control (C) group for peak load.

Similar results were found for stiffness values. Analysis revealed a statistically significant lower stiffness value for the two parallel plates' (2PP) group compared to the spanning plate (SP1) group, the lag screw (LS) group, and the control (C) group. No significant difference was found between the spanning plate (SP1) group, the lag screw (LS) group, and the control (C) group for stiffness. SP2 group was added as an additional group in order to evaluate another potential plating combination with the spanning plate. This group was excluded from the Mann-Whitney statistical analysis comparison in order to avoid confusion over the role of the ladder plate versus the spanning plate in the overall construct strength. The rank order for peak load was (C) 55.8 N > (SP2) 40.6 N > (LS) 34.5 N > (SP1) 32.0 N > (2PP) 7.1 N. The rank order of stiffness was (C) 187 > (SP2) 126 > (SP1) 102 > (LS) 98 > (2PP) 35.

Observations were made regarding fracture pattern of each construct when system failure was reached. The unaltered synthetic mandibles in the control group all fractured in the symphyseal/parasymphyseal region when subjected to the splaying force during mechanical testing. The two parallel plates' (2PP) group showed the least resistance to deformity when tested. In each trial, failure occurred along the symphyseal fracture line with permanent deformation of the plating system or complete failure of the plating system due to fracture of the mandible in a separate location or screw pull-out. This is consistent with similar failure patterns of plate bending and screw pull-out (rather than plate fracture) that have been previously described [9]. In the lag screw (LS) group, failure occurred because of fracture of the synthetic mandible just lateral to where the lag screws penetrated the cortex in the parasymphyseal region. Similarly, failure in the spanning plate groups (SP1 and SP2 groups) occurred by fracture of the synthetic mandible at the fixation point of the spanning plate to the mandible.

## 4. Discussion

Mandible fractures that involve both the parasymphyseal-symphyseal region in combination with single or bilateral subcondylar fracture can lead to widening of the gonial angles if the mandibular arch is insufficiently reduced and stabilized [6]. As noted by Ellis III and Tharanon, particular attention should be given to the reduction and fixation of the buccal cortex in the symphyseal region. “Overreduction” of the fracture with a small gap at the buccal cortex can assure that the lingual cortex is adequately reduced. Overbending of the rigid fixation plate can also be effective. Several authors have investigated the relative strength of different techniques for stabilizing parasymphyseal fractures [7, 10]. The transverse lag screw technique has been found to offer increased stability and resistance to distortion when compared to plates or maxillomandibular fixation (MMF). The transverse lag screw technique can be technically difficult in some types of symphyseal fractures and is also dependent on the skill and experience of the surgeon as well as the availability of adequate length lag screws. We have sought a more universally applicable technique to provide improved stability against the deformational forces involved in this subset of mandible fractures. The use of additional “spanning” miniplate across the inferior border of the anterior mandibular arch in conjunction with traditional parallel upper and lower border plates was felt to be a potential candidate. The advantages of this approach are that it uses readily available plates and screws and is relatively simple to apply. The primary disadvantage is that it requires an external incision in the submental region, while other competing techniques may be performed through an intraoral approach. While this hardware is low profile, it remains potentially palpable, which is a risk with most facial reconstruction hardware. It also requires additional hardware compared to other techniques, which comes with increased financial cost.

The purpose of this study was to compare the biomechanical forces of the standard two parallel plates' (2PP) technique, lag screw (LS) technique, and the spanning plate techniques (SP1 and SP2 techniques). We attempted to mimic the splaying forces across the gonial angles with the use of a synthetic mandible model and servohydraulic mechanical testing unit. The synthetic mandible model has been investigated previously and found to be an adequate substitute for cadaveric mandibles for biomechanical testing purposes [8]. A variety of different constructs have been used for testing the various forces that are in play during mastication. A construct with perpendicular plates for symphyseal fractures has been previously tested [11]; however, this testing was focused on vertical bite forces rather than gonial angle splaying forces. The forces of mastication are varied and extremely complex [12, 13], but, for this particular application (the resistance to deforming forces causing widening of the gonial angles), we felt that direct application of force across the mandibular angles was the most accurate model. We do not represent that our testing construct accurately models all of the physiologic forces that are applied to the mandible during mastication, but it is limited to the splaying forces that we felt most representative of the issue at hand.

As noted in the Results, we found a statistically significant difference in the stiffness values between the lag screw (LS) group and two parallel plates' (2PP) group (97.8 versus 35.1, *P* < 0.009) as well as between the spanning plate (SP1) group and the two parallel plates' (2PP) group (102 versus 35.1, *P* < 0.009) with both the LS and SP1 groups exhibiting increased stiffness compared to the 2PP group. No significant difference was noted between the lag screw group and the spanning plate group (97.8 versus 102, *P* < 0.92). This finding indicates that the spanning plate technique is similar in strength to the lag screw technique. This could also support the use of either of these techniques in the treatment of mandible fractures involving both the symphyseal-parasymphyseal region and one or both subcondylar regions. Although excluded from the Mann-Whitney statistical analysis, the ladder plate and spanning plate (SP2) construct also resulted in higher peak load and stiffness values compared to the two parallel plates plus spanning plate (SP1) and lag screw (LS) groups, indicating another potentially strong construct utilizing a spanning plate.

**Figure 7:**
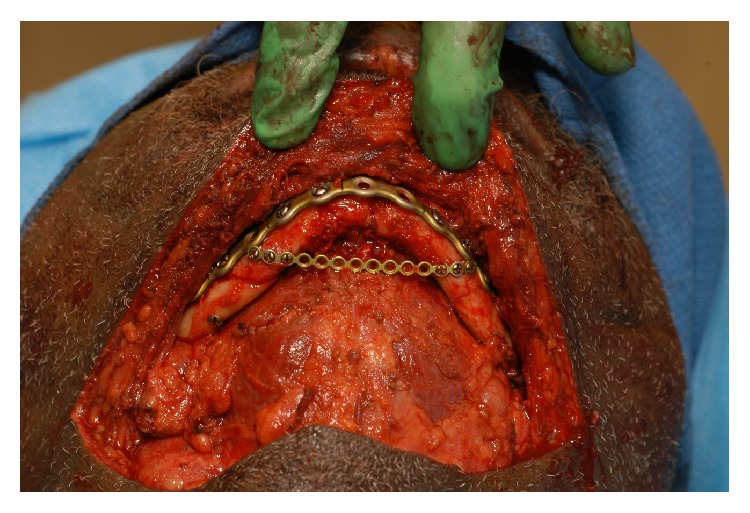
Spanning plate in a selected patient. Note that a different reconstructive plate was used on the anterior mandibular surface due to the multiple comminuted mandibular segments.

The weaknesses of this study include the basic premise that this combination of fractures may lead to splaying of the gonial angles. While this may or may not be a common complication of this particular type of mandible fracture, its occurrence is documented in the report by Ellis III and Tharanon and is supported by the authors' experience [6]. In addition, while this study supports the use of lag screws and spanning plates in this fracture pattern, it may be that more conventional techniques such as properly applied parallel plates with or without MMF may be adequate for reduction and repair of these fractures. We do not advocate the use of the spanning plate techniques in all of these fractures, but when one desires increased stability of the repair, we offer that it may be considered as an alternative to the lag screw technique. Although not specifically described here, we have utilized the spanning plate technique clinically in select cases with positive outcomes (Figure 7).

In summary, the use of a spanning miniplate across the lower border of the anterior mandibular arch appears to offer increased stability to the deformational splaying forces at the gonial angles as compared to the traditional upper and lower parallel plate technique and is at least comparable to the transverse lag screw technique in this synthetic mandible model. Documentation of its use and efficacy in patients with this fracture pattern will require further study.

## Figures and Tables

**Table 1:** Groups.

Group	Fixation technique
Control	No simulated fractures or fixation

2 parallel plates (2PP)	Four-hole 0.6 mm miniplate secured with four 1.7 mm outer diameter screws 4 mm long to upper border of outer cortex + 4-hole 1.5 mm titanium plate secured with four 2.0 mm outer diameter screws 10 mm long to lower border of outer cortex

2PP + spanning plate (SP1)	Four-hole 0.6 mm miniplate secured with four 1.7 mm outer diameter screws 4 mm long to upper border of outer cortex + 4-hole 1.5 mm titanium plate secured with four 2.0 mm outer diameter screws 10 mm long to lower border of outer cortex + 12-hole 1.5 mm titanium plate spanning between the inferior borders of mandibular body secured with four 1.7 mm outer diameter screws 4 mm long

Ladder plate + spanning plate (SP2)	Six-hole 1.5 mm titanium ladder plate secured with four 2.0 mm outer diameter self-tapping screws 10 mm long to outer cortex + 12-hole 1.5 mm titanium plate spanning between the inferior borders of mandibular body secured with four 1.7 mm outer diameter self-tapping screws 4 mm long

Lag screw (LS)	Two 30 mm long 2.0 mm outer diameter self-tapping screws

**Table 2:** Summary of results (mean).

Group	Peak load (N)	Stiffness (force versus extension)
Control	55.8	187
2 parallel plates (2PP)	7.1	35.1
2PP + spanning plate (SP1)	32.1	102
Ladder plate + spanning plate (SP2)	40.6	126
Lag screw (LS)	34.5	97.8

**Table 3:** Statistical analysis summary.

Test	Between groups	Statistical significance	*P* value
Peak load	Control versus 2PP	Yes	0.004
Peak load	Control versus SP1	No	0.04
Peak load	Control versus LS	No	0.09
Peak load	2PP versus SP1	Yes	0.009
Peak load	2PP versus LS	Yes	0.009
Peak load	SP1 versus LS	No	0.92
Stiffness	Control versus 2PP	Yes	0.004
Stiffness	Control versus SP1	No	0.01
Stiffness	Control versus LS	No	0.01
Stiffness	2PP versus SP1	Yes	0.009
Stiffness	2PP versus LS	Yes	0.009
Stiffness	SP1 versus LS	No	0.92

*P* < 0.01 considered statistically significant.
